# Evaluation of indigenous *Trichoderma* isolates from Manipur as biocontrol agent against *Pythium aphanidermatum* on common beans

**DOI:** 10.1007/s13205-011-0027-3

**Published:** 2011-10-13

**Authors:** Th. Kamala, S. Indira

**Affiliations:** Microbial Resources Division, Institute of Bioresources and Sustainable Development (IBSD), Autonomous DBT Research Institute, Government of India, Takyelpat, Imphal, 795001 Manipur India

**Keywords:** Biological control, *Pythium aphanidermatum*, *Trichoderma* isolates

## Abstract

*Pythium aphanidermatum* is one of the common causal pathogen of damping-off disease of beans (*Phaseolus vulgaris* L.) grown in Manipur. A total of 110 indigenous *Trichoderma* isolates obtained from North east India were screened for their biocontrol activity which can inhibit the mycelial growth of *P. aphanidermatum*, the causal organism of damping-off in beans. Out of the total isolates, 32% of them showed strong antagonistic activity against *P. aphanidermatum* under in vitro condition and subsequently 20 best isolates were selected based on their mycelial inhibition capacity against *P. aphanidermatum* for further analysis. Different biocontrol mechanisms such as protease, chitinase, *β*-1,3-glucanase activity, cellulase and production of volatile and non-volatile compounds were also assayed. Based on their relative biocontrol potency, only three indigenous *Trichoderma* isolates (T73, T80 and T105) were selected for pot culture experiment against damping-off diseases in common beans. In greenhouse experiment, *Trichoderma* isolates T-105 significantly reduced the pre- and post-emergence damping-off disease incidence under artificial infection with *P. aphanidermatum* and showed highest disease control percentage.

## Introduction

Modern agriculture is highly dependent on the use of chemical pesticides to control plant pathogens. Fungicides and fumigants commonly have drastic effects on the soil biota, as they are intentionally applied at much higher rates than herbicides and insecticides (Fraser 1994). These methods pollute the atmosphere, and are environmentally harmful, as the chemicals build up in the soil (Nannipieri 1994). Furthermore, the repeated use of such chemicals has encouraged the development of resistance among the target organisms (Goldman et al. 1994). Therefore, control of plant pathogens using microbial bioinoculants has been considered as a potential control strategy in recent years. For instance, integration of biocontrol agents with reduced doses of chemical agents has the potential to control plant pathogens with minimal impact on the environment (Chet and Inbar 1994). Therefore, search for these biological agents is increasing.

Beans (*Vicia faba* L.) is an important winter vegetable legume of North east India and considered as a meat and a skim milk substitute in diet for its high protein and nutritional quality (Chavan et al. 1989). *Pythium* damping-off is one of the major factors limiting the production of certain field crops in North east India. It is one of the major disease problems hampering the production of beans in this region. *Pythium* spp. are worldwide in distribution (Hendrix and Campbell 1973) that attack all stages of the various crops causing significant losses in yield. *Trichoderma*, a saprophytic fungus is known to be one of the best candidate of biocontrol agents. Modes of action of this fungus include mycoparasitism, antibiosis, competition for nutrients and space, tolerance to stress through enhanced root and plant development, solubilization and sequestration of inorganic nutrients, induced resistance, and inactivation of the pathogen’s enzymes (Scala et al. 2007). The antagonistic action of *Trichoderma* species against phytopathogenic fungi might be due to either by secretion of extracellular hydrolytic enzymes (Chet 1987; Di Pietro et al. 1993; Schirmbock et al. 1994) or by the production of antibiotics (Dennis and Webster 1971a, b; Claydon et al. 1987). They are known to be the most commonly used antagonists against *Pythium aphanidermatum* as they have more than one mechanism of action (Chet et al. 1981; Hadar et al. 1984; Krishnamurthy 1987; Kumar and Mukhopadhyay 1994, Shanmugam and Varma 1999; Hazarika et al. 2000; Manoranjitham et al. 2001). It also produces a large variety of volatile secondary metabolites such as ethylene, hydrogen cyanide, aldehydes and ketones which play an important role in controlling the plant pathogens (Vey et al. 2001). Although, exotic strains may be effective in diseases control, but, molecular technique-based evidence suggests that genotypes of beneficial microorganisms may be endemic to a biogeographical region (Cho Jae-Chang and Tiedji 2000). The endemic microbial pool of a region may contain highly efficient genotypes and is likely to perform better than the exotic strains. Due to the occurence of rich biodiversity in the Indo-Burma Biodiversity hot spot region, Manipur is likely to harbour useful *Trichoderma* isolates. So far, not much previous research has been done on the use of microbial agents for the control of damping-off diseases of beans in this region. Therefore, the present study describes the impact of different indigenous *Trichoderma* isolates in controlling of *P. aphanidermatum* the causal agent of damping-off disease in in vitro as well as in vivo conditions.

## Materials and methods

### Isolation of *Trichoderma* spp.

Soil samples were collected from agricultural fields of Imphal East and West districts of French bean growing fields of Manipur using TSM (*Trichoderma* selective medium) by the following modified method given by Tate (1995). Then, the colonies were transferred on PDA plates and incubated at 27 °C for 5–6 days followed by morphological identification based on colony characteristics (Gams and Bisset 1998) and microscopically according to the related literatures and identified on the basis of cultural and microscopic morphological characters (Barnett and Hunter 1972; Bissett 1991) using trinocular microscope.

## Evaluation of antagonistic activity of *Trichoderma* species

The antagonistic effect of 110 *Trichoderma* isolates was evaluated against *P. aphanidermatum* in in vitro condition using dual culture technique **(**Coskuntuna and Özer 2008). Each *Trichoderma* spp. and *P. aphanidermatum* were cultured, separately, on PDA medium for 7 days at 25 °C. 4-day-old *Trichoderma* cultures were inoculated on one site of PDA plate and *P. aphanidermatum* cultures were inoculated at the opposite side of Petri dish and plates were incubated at 25 °C for 7 days. The colony interaction was assayed as percentage inhibition of the radial growth of the *Trichoderma* spp. towards the opponent antagonist using the following formula (Fokkema 1976):where, *A* is the diameter of mycelial growth of pathogenic fungus in control and *B* is the diameter of mycelial growth of pathogenic fungus with *Trichoderma* isolate.

### In vitro characterization of different biocontrol mechanisms of *Trichoderma* isolates

#### Production of volatile and non-volatile metabolites

Twenty selected *Trichoderma* isolates based on the mycelium inhibition assay against *P. aphanidermatum* were evaluated for the production of volatile inhibitory substances in in vitro conditions following the modified methods of Dennis and Webster (1971a). 5-mm disc of *Trichoderma* colony was inoculated centrally in petriplates containing PDA medium in triplicates. The petri plates were sealed at the edges and incubated at room temperature. After 5 days, the test pathogens were inoculated on fresh PDA and the lids of the petriplates inoculated with antagonist were replaced by the pathogen on PDA. The plates were fixed with cellophane-tape and incubated for another 7 days; whereas, control plates were inoculated with pathogen alone. Growth of *P. aphanidermatum* was measured after 5–6 days of incubation and the inhibition zones were recorded.

The production of non-volatile substances by the *Trichoderma* isolates against the test pathogen was studied using the method described by Dennis and Webster (1971b). *Trichoderma* isolates were inoculated in 100 ml sterilized potato dextrose broth (PDB) in 250 ml conical flasks and incubated at 25 ± 1 °C on a rotatory shaker set at 100 rpm for 15 days. The control flasks were not inoculated with any of the culture. The culture was filtered through Whatmann filter paper for removing mycelial mats and then sterilized by passing through 0.2 μm pore biological membrane filter. The filtrate was added to molten PDA medium (at 40 ± 3 °C) to obtain a final concentration of 10% (v/v). The PDA containing Petri dishes was inoculated with 5 mm mycelial plugs of the pathogens at the centre and plates were incubated at 25 ± 1 °C for 3 days or until the colony reached the plate edge. There were three replicates for each treatment. The inhibition zone of the mycelial growth in relation to growth of the controls was recorded.

#### Production of protease enzymes

Protease activity of *Trichoderma* isolates was determined using Skim milk agar medium (51.5/l) (Berg et al. 2002). Culture disc from 5- to 6-day-old *Trichoderma* cultures was inoculated on skim milk agar medium and incubated at 28 °C ± 2 °C for 3–4 days. *Trichoderma* with proteases activity gave a clearance zone around the colony indicating the production of protease enzymes.

#### Production of chitinase activity

Chitinase activity of the *Trichoderma* isolates was determined on chitin detection medium (Roberts and Selitrennikoff (1988).

*Preparation of colloidal chitin*: 5.0 g of chitin was added to 60 ml of conc. HCl (acid hydrolysis) by constant stirring using a magnetic stirrer at 4 °C and kept in refrigerator overnight. The resulting slurry was then added to 200 ml of ice-cold 95% ethanol and kept at 26 °C overnight (ethanol neutralization). Then it was centrifuged at 3,000 rpm for 20 min at 4 °C. The pellet was repeatedly washed with sterile distilled H_2_O by centrifugation at 3,000 rpm for 5 min at 4 °C until the smell of alcohol vanished. The final colloidal chitin was stored at 4 °C until further use.

*Chitinase detection medium*: The final chitinase detection medium per litre comprises 4.5 g colloidal chitin, 0.3 g magnesium sulphate, 3.0 g ammonium sulphate, 2.0 g potassium dihydrogen phosphate, 1.0 g citric acid monohydrate, 15 g agar, 0.15 g bromocresol purple and 200 μl of tween-80. The pH of the media was maintained at 4.7 and autoclaved at 121 °C for 15 min. The fresh culture plugs of *Trichoderma* isolates to be tested for chitinase activity were inoculated into the sterile plates containing chitinase detection medium and incubated at 28 ± 2 °C for 2–3 days and observed for the coloured zone formation. Chitinase activity was identified due to the formation of purple coloured zone. The principle behind the formation of coloured zone is that the media is supplemented with a pH indicator dye, bromocresol purple which transforms the yellow colour of the media (in acidic here pH 4.7) into purple colour due to increase in pH. The pH increases because of the utilization of chitin by the *Trichoderma* and its breakdown into product *N*-acetyl glucosamine. Colour intensity and diameter of the purple coloured zone were taken as the criteria to determine the chitinase activity after 3 days of incubation.

#### Production of β-1,3-glucanases

For plate screening of β-1,3-glucanases activity, carboxy methyl cellulose agar (CMC agar) medium amended with laminarin was used according to the modified method given by El-Katatny et al. 2001. 1 l of CMCA contained 1 g of ammonium dihydrogen phosphate, 0.2 g of potassium chloride, 1 g of magnesium sulphate, 1 g of yeast extract and 1,000 ml of distilled water. A 6 mm culture disc was placed at the centre of the plate. Plates were incubated at 25 °C for 3 days. β-1,3-glucanases activity on the plates was observed by dipping in 0.1% congo red dye for 15–20 min followed by distaining with 1 N NaCl and then with 1 N NaOH for 15 min. The distaining was repeated twice. β-1,3-glucanase activity was recorded with the clearance zone formation.

#### Production of cellulase activity

To determine the production of cellulase activity from the *Trichoderma* isolates, 5 mm disc of *Trichoderma* cultures was inoculated on Czapek-mineral salt agar medium. 1 l of Czapek-mineral salt agar medium consist of 2.0 g of NaNo_3_, 1.0 g of K_2_HPO_4_, 0.5 g of MgSo_4_.7H_2_0, 0.5 g of KCl, 5.0 g of CMC, 2.0 g of peptone, 20.0 g of agar and 1,000 ml of distilled H_2_O. Inoculated plates were incubated at 35 °C in an inverted position. After 2–5 days, the plates were flooded with 1% aqueous solution of hexadecyltrimethyl ammonium bromide. The degree of cellulase activity production was observed according to the formation of a clearance zone formation around the colony (Aneja 2004).

After assessing the biocontrol properties exhibited by the selected 20 *Trichoderma* isolates under in vitro condition, only three isolates, T73, T80 and T105 were selected for further pot experiment under greenhouse conditions.

## Antagonistic activity of the selected *Trichoderma* isolates in green house conditions

Based on various biocontrol potency exhibited by the indigenous *Trichoderma* isolates, three isolates viz. T73, T80 and T105 were selected for pot culture experiments against *P. aphanidermatum* under artificially infestation conditions. The experiment was designed under greenhouse conditions at IBSD, Imphal during May–August for 2 consecutive years in 2009–2010. Each pot was taken with 4 kg of sterilized, loamy, clay soil. 2 days before seedling, soil was infested with *P. aphanidermatum* at the rate of 5 g/kg soil. Simultaneously after 2 days of pathogen inoculation, soils were inoculated with *Trichoderma* isolates at 5 g/kg soil, and then pots were watered for 7 days before sowing. Six bean seeds were sown in each pot. Three pot replicates for each treatment. Pots were kept under greenhouse conditions till the end of the experiment. Disease incidence of pre-and post-emergence and survival (%) of bean plants were recorded after 15, 30 and 45 days, respectively. The percent disease incidence was calculated using the following formula.

## Statistical analysis

Data obtained from all the experiments were analysed by analysis of variance ANOVA using SPSS Statistical Package. List significance difference (LSD) at 5% level of significance (*P* = 0.05) was used to compare the mean values of different treatments in the experiment.

## Results

### In vitro antifungal activity

**Fig. 1:**
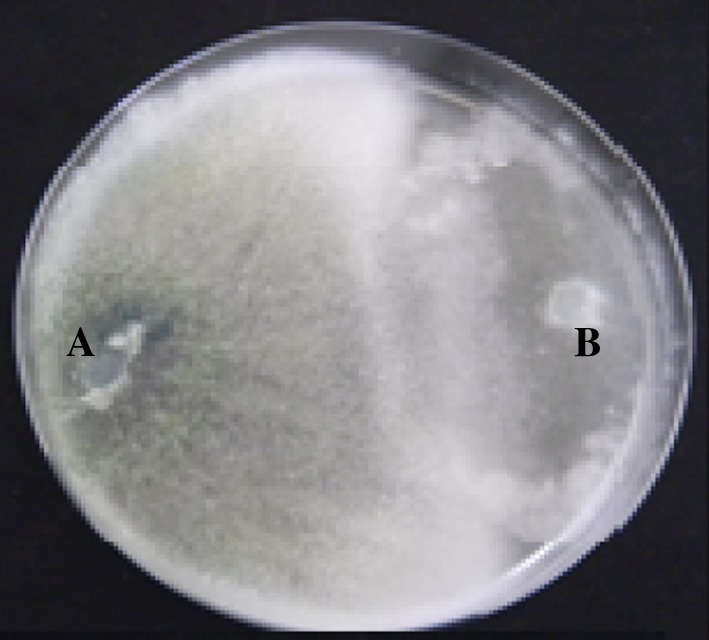
Antagonistic activity of the *Trichoderma* islolate against the *P. aphanidermatum.***a***Trichoderma* isolate, **b***P. aphanidermatum*

Out of the total *Trichoderma* isolates tested, 32% of them shows antifungal antagonistic activity in in vitro conditions against *P. aphanidermatum* the causal agents of damping-off disease in beans. Based on the dual plate assay results of mycelial growth inhibition, 20 potential *Trichoderma* isolates were selected for further characterization (Table 1; Fig. 1). Six isolates could significantly inhibit the external growth of *P. aphanidermatum* with the highest disease reduction percentage. The inhibition % was in the range of 4.16–84%. Maximum inhibition zone was exhibited by T105 (84%) while the lowest one was observed in T71 (4.16%) (Table 1).

**Table 1:** In vitro antifungal activity of the selected *Trichoderma* isolates against *P. aphanidermatum*

Sl. no.	*Trichoderma* isolates	Antifungal activity (inhibition zone in mm) *Pythium aphanidermatum*	Disease incident %	Production of metabolites	
				Volatile metabolites	Non-volatile metabolites
1	T11	71.00 ± 2.08a	11.25	+	–
2	T21	48.33 ± 1.67c	19.58	–	–
3	T24	76.33 ± 1.86a	15.41	–	–
4	T37	42.33 ± 1.45c	27.08	–	–
5	T39	67.67 ± 1.45b	15.41	–	–
6	T41	62.67 ± 1.45b	21.66	–	–
7	T51	71.67 ± 0.89a	10.41	–	–
8	T61	70.00 ± 1.15a	12.50	–	–
9	T66	46.00 ± 1.00c	42.5	–	–
10	T68	49.00 ± 1.50c	38.75	–	–
11	T70	67.00 ± 1.08b	16.25	–	+
12	T71	12.33 ± 1.45d	84.59	–	–
13	T73	75.67 ± 1.20a	5.41	+	+
14	T77	48.33 ± 2.02c	39.59	–	–
15	T80	72.33 ± 1.45a	9.59	–	–
16	T83	42.33 ± 1.45c	47.09	–	+
17	T86	64.33 ± 2.33b	19.59	–	−
18	T89	40.00 ± 1.15c	50.00	+	+
19	T100	65.33 ± 1.45b	18.34	–	–
20	T105	76.67 ± 1.67a	4.16	+	+
21	Control	80.00 ± 0.12a	0	–	–
LSD (*P* = 0.05)		8.73			

Values are average of three replicates ± SEM

Values in the column followed by same letter are not significantly different (*P* < 0.05)

### Production of volatile and non-volatile metabolites

After 7 days of incubation, the production of secondary metabolites was observed and it was found that only four *Trichoderma* isolates (T11, T73, T89 and T105) exhibited the production of volatile metabolites with maximum growth inhibition of *P. aphanidermatum* when compared to the other treatment; while, five *Trichoderma* isolates (T70, T73, T83, T89 and T105) showed the production of non-volatile compounds by inhibiting the growth of the *P. aphanidermatum* (Table 1).

### Determination of different biocontrol enzymatic activity

The production of different types of hydrolytic enzymes by the selected 20 *Trichoderma* isolates was assayed. Results were recorded according to the diameter in mm produced in the enzyme detection plates.

**Fig. 2:**
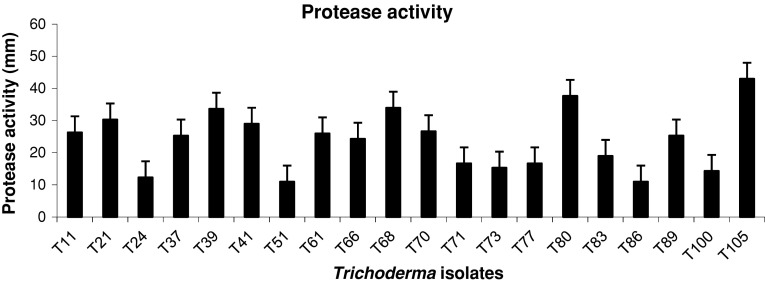
Specific activity of protease from different selected *Trichoderma* isolates. (Each *bar* represents the average of three independent measurements)

The protease activities produced by the different *Trichoderma* isolates range from 11 to 43 mm in diameter. The maximum activity was recorded in T105 (43 mm) followed by T80 (37.67 mm) and T68 (34 mm) while the least activity was recorded in T51 (11 mm) (Fig. 2).

**Fig. 3:**
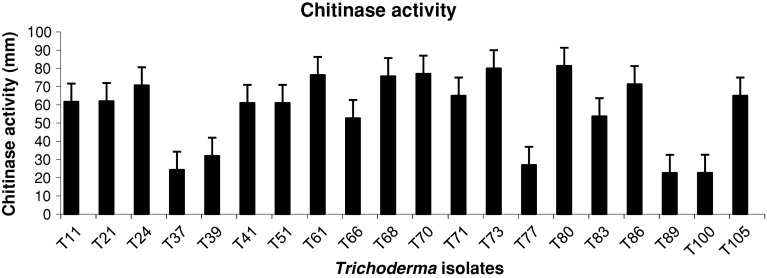
Specific activity of chitinase from different selected *Trichoderma* isolates. (Each *bar* represents the average of three independent measurements)

With the degree of the formation of purple colour zone on the chitin detection medium, the production of chitinase activity has been recorded. The chitinase activity of the selected 20 *Trichoderma* isolates varied from one another (Fig. 3). The highest activity was recorded in the case of T80 with a zone diameter of 81.33 mm followed by T73 (80 mm) and T61 (76.33 mm), while T89 produced minimum chitinase activity producing only 22.6 mm clearing zone diameter.

**Fig. 4:**
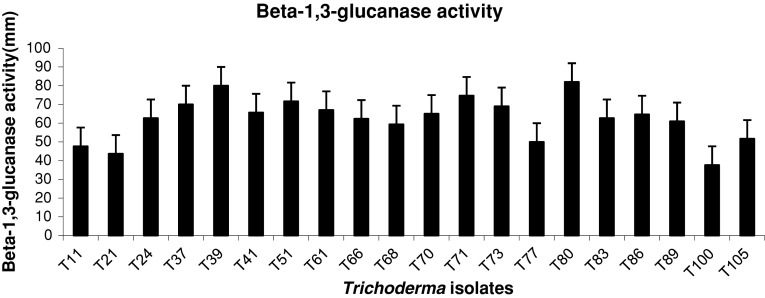
Specific activity of β-1,3-glucanase activity from different selected *Trichoderma* isolates. (Each *bar* represents the average of three independent measurements)

The data in Fig. 4 showed that T80 significantly produced maximum β-1,3-glucanase activity with 82 mm zone diameter which was followed by T39 (80 mm) and T73 (69 mm). Among the tested isolates, the least β1,3-glucanase activity was recorded in case of T100 (37.66 mm).

**Fig. 5:**
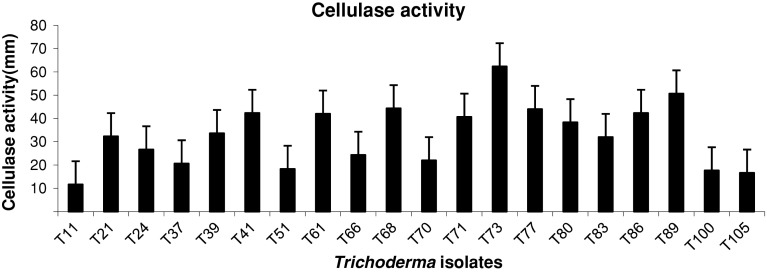
Specific activity of cellulase activity from different selected *Trichoderma* isolates. (Each *bar* represents the average of three independent measurements)

It is evident from the data in Fig. 5 that the highest cellulase activity was observed in case of T73 (62.33 mm), which was followed by T105 (50.67 mm) and T68 (44.33). The least cellulase activity was produced by T11 (11.67 mm).

### In greenhouse experiment

**Fig. 6:**
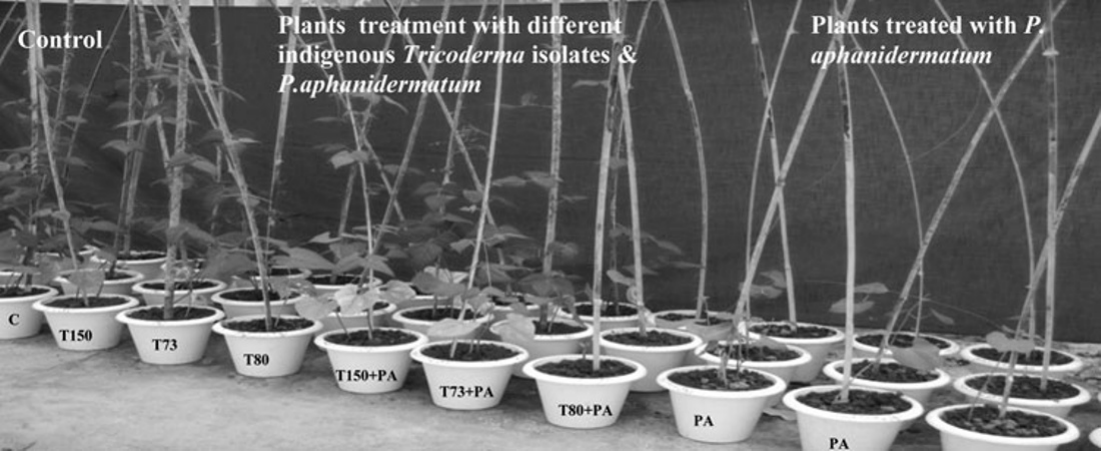
Pot experiment of *Trichoderma* biocontrol efficacy in beans against *P. aphanidermatum* under greenhouse condition

In controlled environmental conditions, soil treatments with T73, T80 and T105 significantly (*P* = 0.05) reduced the pre- and post-emergence damping-off disease incidence under artificial infection with *P. aphanidermatum* in greenhouse conditions (Table 2). The damping-off disease incidence at the pre-emergence caused by combined application of *P. aphanidermatum* and *Trichoderma* spp. was in the range of 9–22%. After 30 days of plantation, the growth patterns were observed and recorded. At this stage, T105 gave the highest reduction to disease incidence by 82.86%, followed by T73 (63.81%), and T80 (22.0%). At post-emergence stage, the disease incidence ranges from 5.8 to 8.2%. T105 gave the highest growth reduction of 90.72% to disease incidence followed by T73 (88.00%) and T80 (86.88%), respectively. The percentages of survival bean plants were in the range of 78.5–90.1 compared to 42.4% healthy bean plants in the control treatment. T105 gave 90.1% healthy plants, followed by T73 (87.2%), and T80 (78.5%), respectively (Table 2; Fig. 6). From this data, it is clear that the indigenous T105 isolates have a good potentiality of controlling the damping-off disease of beans caused by the *P. aphanidermatum*.

**Table 2:** Effect of *Trichoderma* isolates treatments on the percentage of damping-off disease of bean plants under greenhouse condition (artificial infection)

*Trichoderma* isolates	Disease assessment				Survival
	Damping-off in beans (by *Pythium aphanidermatum*)				Healthy plants (%)
	Pre-emergence		Post-emergence		
	Incidence (%)	Reduction (%)	Incidence (%)	Reduction (%)	
T73	22.0b	58.09	7.5c	88.00	87.2b
T80	19.0c	63.81	8.2b	86.88	78.5c
T105	9.0d	82.86	5.8d	90.72	90.1a
Control	52.5a		62.5a		42.2d

Means in each column followed by the same letter are not significantly different according to LSD test (*P* = 0.05)

## Discussion

An important finding of the present study is the identification of indigenous *Trichoderma* isolates from the unique biodiversity hot spot zone of North east India as potential biocontrol agents. The results obtained indicate that the selected *Trichoderma* isolates can be a potential source of biocontrol agents for the control of damping-off disease in beans grown in this region which may be due to location specific and well adaptation with the existing environmental conditions of the region and also due to pathogen specificity. Maximum inhibition of the pathogen was observed with T105 (76.67 mm). Formation of inhibition zone against *P. aphanidermatum* in dual cultures could be explained on the basis of production of extracellular hydrolytic enzymes by *Trichoderma* (El-Katatny et al. 2001). Fajola and Alasoadura (1975) found that culture filtrates of *T. harzianum* inhibit zoospore germination, germ tube elongation and mycelial growth of *P. aphanidermatum* causing the damping-off disease of tobacco. Formation of inhibition zone at the contact between *Trichoderma* and *P. aphanidermatum* in dual cultures could be explained on the basis of production of volatile and non-volatile metabolites as well as the production of extracellular hydrolytic enzymes by *Trichoderma* (El-Katatny et al. 2001).

In the present study, we tested local *Trichoderma* isolates for the production of volatile and non-volatile compounds that can inhibit the growth of *P. aphanidermatum* in in vitro condition. From the result, it is evident that volatile compounds produced by *Trichoderma* suppress the mycelial growth of *P. aphanidermatum* and found effective when compared to the other treatments. The earlier studies also revealed that antimicrobial metabolites produced by *Trichoderma* are effective against a wide range of fungal phytopathogens e.g., *Fusarium oxysporum*, *Rhizoctonia solani, P. aphanidermatum, Curvularia lunata, Bipolaris sorokiniana* and *Colletotrichum gloeosporiodes* (Xiao-Yan et al. 2006; Zivkovic et al. 2010).

The extracellular enzymes produced by *Trichoderma* isolates may be correlated with the antagonism. Results from the current study showed that most of the selected 20 *Trichoderma* isolates have given good enzymatic activities. Elad et al. (1982) reported that the isolates of *T. harzianum*, which were found to differ in their ability to attack *Sclerotium rolfsii, Rhizoctonia solani* and *P. aphanidermatum*, also differed in the levels of mycolytic enzymes produced by them. *Trichoderma* directly attacks the plant pathogen by excreting lytic enzymes such as chitinases, β-1,3-glucanases, proteases and cellulases (Elad et al. 1982; Haran et al. 1996) and also with the production of volatile and non-volatile metabolic compounds. In the present study, T105, T80. T80 and T73 isolates were observed to be the efficient producer of proteases, chitinases, β-1,3-glucanases and cellulases activity, respectively. Lorito et al. (1994) have shown the involvement of glucanases in mycoparasitism. Involvement of proteases in biocontrol processes has already been reported (Elad and Kapat 1999; Pozo et al. 2004). De marco and Felix (2002) observed that the biocontrol potential of an Indian *Trichoderma* isolates against *C. perniciosa* was due to protease activity. In the present study, T73, T89 and T86 were observed to be the efficient producer of cellulases enzymes. This might be one of the reasons for its biocontrol potentiality. As the cell wall of *Pythium* species is composed of cellulose and 1 β-1,3-glucan (Bartinicki-Garcia 1968), the enzymes produced by *Trichoderma* might be involved in hydrolysis of *P. aphanidermatum* cell wall during antagonism (Thrane et al. 1997). Lorito et al. (1994) reported the involvement of glucanases in mycoparasitism.

Our results revealed that the T73, T80 and T105 isolates, which were obtained from the rhizosphere soil of healthy bean plants, have been reported to have strong biocontrol activity against damping-off disease caused by *P. aphanidermatum* under in vitro as well as pot culture experiment thereby reducing the mycelial growth of the pathogenic fungi. Since the continuous use of chemical methods is not economical in long run thereby polluting the environment leaving harmful residues which may lead to development of resistant strains among the target organisms. Therefore, the above overall results indicated that the application of indigenous *Trichoderma* isolates in the pot experiment tended to reduce the incidence of pre- and post-emergence of damping-off disease of the beans (Table 2). These results agree with those recorded by Thrane et al. 1997**.** Biocontrol agents such as T73, T80 and T105 isolates used in the present study, may better be able to colonize the rhizosphere by inhibiting the pathogen community (Bennett and Whipps 2008b). Our results, based on soil treatments with the tested *Trichoderma* isolates demonstrated an effective reduction of incidence of damping-off disease in bean caused by *P. aphanidermatum* under in vitro as well as the pot experiment (El-kafrawy 2000; Gonzalez et al. 2005; Malik et al. 2005).
